# Heat Generation and Efficiency of a New Modified Phaco Tip and Sleeve

**DOI:** 10.1371/journal.pone.0159049

**Published:** 2016-08-03

**Authors:** Aeri Yoo, Ki Yeun Nam, Hungwon Tchah, Myoung Joon Kim

**Affiliations:** 1 Department of Ophthalmology, Saevit Eye Hospital, Goyang, Korea; 2 Department of Physical Medicine and Rehabilitation, Dongguk University College of Medicine, Goyang, Korea; 3 Department of Ophthalmology, University of Ulsan College of Medicine, Asan Medical Center, Seoul, Republic of Korea; National Eye Institute, UNITED STATES

## Abstract

**Purpose:**

To compare a modified phacoemulsification tip with the established micro tip, in terms of temperature at the corneal wound, efficiency, and chatter events, using the Centurion^®^ Vision system.

**Methods:**

Eighty porcine eyes were randomized into 4 groups: 1)sleeveless conventional 45D MiniFlared ABS^®^ Kelman tip (1.1-mm incision); 2)sleeveless new modified 45D ABS^®^ INTREPID^®^ balanced tip(1.1-mm incision); 3) Kelman tip with own sleeve (2.2-mm incision); 4)Balanced tip with modified 4-rib sleeve (2.2-mm incision). Measurements were taken with 2 settings: longitudinal(power 40% and 70%) and torsional mode (power 40% and 100%). Peak temperatures were measured 0, 10, 30, and 60 seconds after continuous ultrasound power. For the efficiency test, porcine lens nuclei were formalin soaked and cut into 2.0 mm^3^ cubes. Efficiency and chatter were examined.

**Results:**

In all longitudinal settings, the sleeveless groups(1 and 2) showed lower temperatures than the sleeved groups(3 and 4) (P = 0.003). In 100% torsional mode, groups 3 and 4 produced significantly different temperatures(37.13 ± 1.44 and 35.14 ± 0.54, respectively; P = 0.007).The efficiency, in a 100% power torsional setting, was13.52 ± 2.60 sec for group 4, and 44.45± 14.75 sec for group 3 (P<0.001).

**Conclusions:**

The two different bare tips show no significant differences in thermogenesis. However, the balanced tip with sleeve produces lower temperaturesat100% torsional power and better efficiency than the Kelman tip.

## Introduction

**Fig 1:**
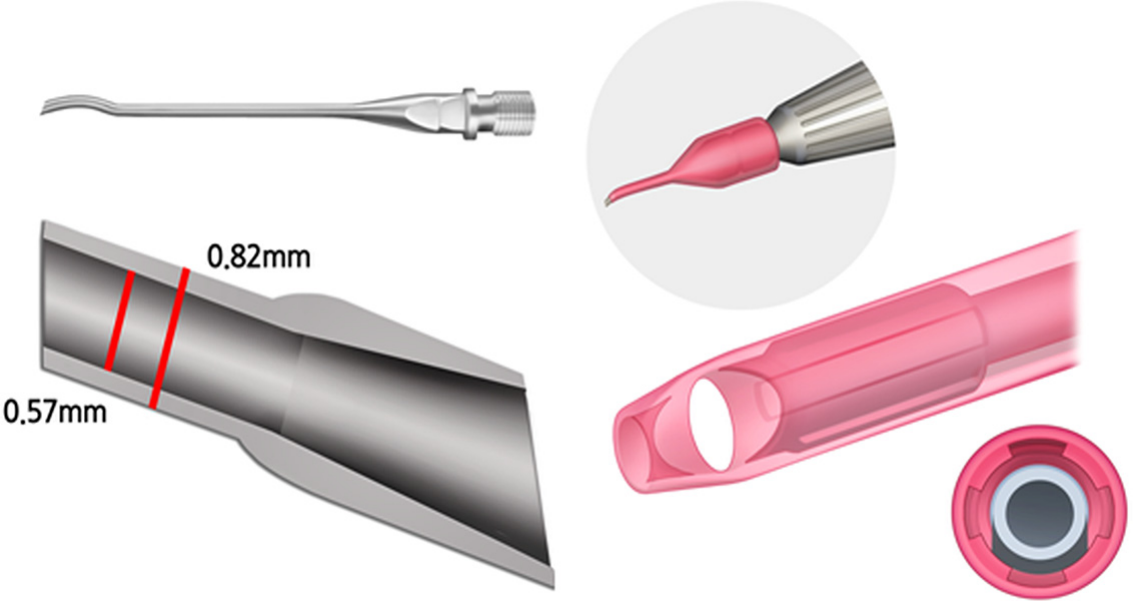
New modified phaco tip and sleeve. *Left*: Balanced tip has same inner caliber as Kelman tip, however, its thickness decrease using titanum material. *Right*: Sleeve has been modified with 4 rib- like space as well.

As methods for performing cataract surgery have improved from intracapsular cataract extraction to microincision cataract surgery (MICS), the incision size has decreased. MICS, with incisions ranging from 1.6 to 2.2 mm, is becoming increasingly popular because it is often associated with many advantages, such as the use of a lower effective phaco power,[1] a reduction in surgically induced astigmatism, and faster refractive stability.[2] Reduction of the incision size can still generate significant thermal energy at the incision site and cause corneal burns[3]. This thermal generation is one of the important factors to consider in the development of new phaco tips. Recently, Alcon Surgical(Fort Worth, TX) launched a new phaco machine, Centurion®, with anew phaco tip with modified sleeve. Newly released Balanced tip has same inner caliber as Kelman tip, however, its thickness decrease using titanum material. The shape of Balanced tip has been changed from straight to bent form. Sleeve has been modified with 4 rib- like space as well (Fig 1).

The primary goal of our present study was to evaluate the temperature profiles at the corneal wound with two different phaco tips (balanced versus Kelman tip) and 2 different sleeves. The secondary goal of this study was to evaluate the efficiency and chatter events with 2 different phaco tips using the Centurion^®^ Vision system.

## Materials and Methods

### Experiment 1 (Temperature profile)

Freshly harvested eighty porcine eyes obtained from a local slaughterhouse. This porcie eyes and study was reviewed and apporoved by the Institutional Animal Care and Use Committee (IACUC) Asan Institute for Life Sciences, Asan Medical Center. The committee abides by the institute of Laboratory Animal Resources (ILAR) guide.)

Eighty postmortem porcine eyes were kept at room temperature(~20°C) for approximately 2 hours before surgery. Eyes were randomized into 4 groups, as follows. Group 1: conventional 45D MiniFlared ABS^®^ Kelman tip (kelman tip) without sleeve (1.1-mm incision); group 2: new modified 45D ABS^®^ INTREPID^®^ Balanced tip (balanced tip) without sleeve (1.1-mm incision); group 3: Kelman tip with 0.9mm Microsmooth Ultra sleeve (microsmooth sleeve) (2.2-mm incision); group 4: Balanced tip with modified 4-rib sleeve design INTREPID^®^ sleeve (intrepid sleeve)(2.2-mm incision). Measurements were taken in 2 settings: longitudinal mode (power 40% and 70%) and torsional mode (power 40% and 100%). To capture the heat emanating from the wound, an IR thermal-imaging camera (FLIR i7,FLIR Systems, Inc.) was focused on the wound and images were captured at 7 frames per second. Peak temperatures were measured 0, 10, 30, 60 seconds after continuous ultrasound power. The fluid settings were the following: vacuum level 200 mm Hg, aspiration rate 20 cc/min, and bottle height 80 cm. Balanced salt solution was used as irrigating fluid. Corneal burns were defined by clinical observation of localized corneal whitening by 2 observers.

### Temperature recording

The incision wound surfaces were imaged in the infrared spectrum using an IR thermal-imaging camera (FLIR i7, FLIR Systems, Inc.). All objects emit infrared radiation as a function of temperature. A thermal camera captures the infrared radiation emitted from an object surface and converts it to temperature readings. In the experiments, the camera was set to display temperatures between 20°C and 90°C (dynamic range of camera: -40°C‒120°C). Camera calibration was verified by measuring warm- and cold-water baths using the infrared camera and then comparing these readings with those from a thermocouple at temperatures across the experimental range.

### Experiment 2 (efficiency and chatter)

**Fig 2:**
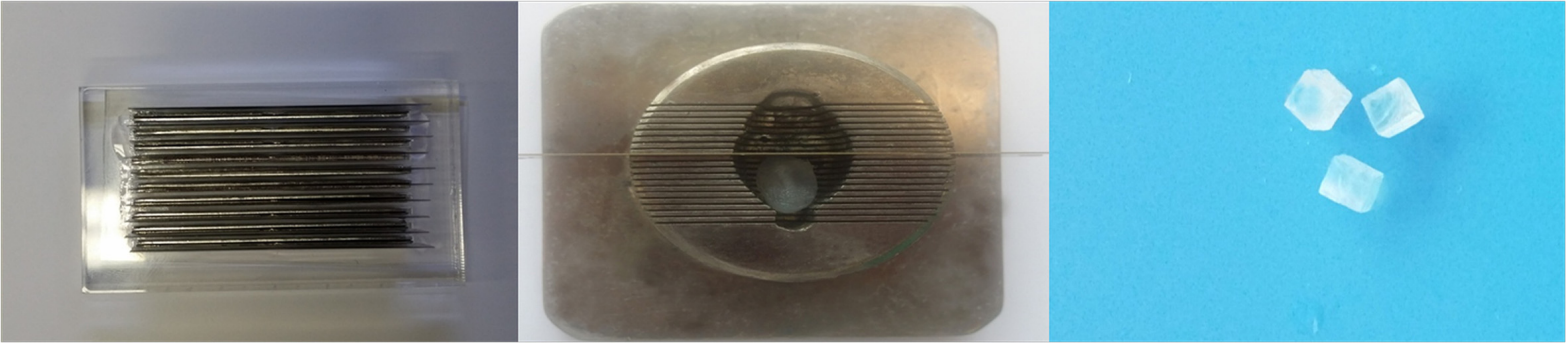
Porcine lens cutting methods. *Left*: Own cutting knife was made with acryl plate and laser cutter with 2mm interval. *Middle*: The lens was trapped between the blades, cutting knife were inserted plate slots. Right: The porcine lens is cut into 2.0 mm^3^ cubes.

Whole porcine lens dissection and experiments were conducted within 24–48 hours after procurement. Porcine lens nuclei were prepared according to previously published methods.[4].[5,6] Briefly, the nuclei were isolated from porcine eyes and placed in balanced salt solution (BSS) for approximately 1 hour, until all lenses were removed from the globes. Lens nuclei were then fixed individually in 10 mL of 10% normal buffered formalin for 24 hours at room temperature. One modification of the standard procedure was that we fixed the porcine lenses for 24 hours in order to harden the nucleus. After fixation, the lenses were washed 3 times with BSS. The lenses then were allowed to equilibrate in BSS for less than 48 hours at room temperature. Multiple lens-cutting designs were tested to create consistent cubes. Finally, our own cutting knife was made with acryl plate and laser cutting and engraving machine BCL-X series machine (Bodor CNC Machine CO.,Ltd, Jinan, China). On the acryl plate, we made fine cuts by 2mm intervals, in which sharp blades were inserted. The cutter sliced a piece of nuclei. which was held in position in a brain matrix(Ted Pella, Inc. USA) by a blade. The porcine lenses were cut into 2.0mmx2.0mmx2.0mm cubes with our own cutting knife and (Fig 2).

Phacoemulsification of the individual lens cubes was performed using Centrion®, the Ozil Intelligent Phaco (IP) machine (Alcon Surgical, Fort Worth, Texas). We used a constant vacuum(550 mm Hg), constant aspiration rate (40 mL/min), and constant bottle height (50 cm) with an intelligent phacoemulsification setting.[7] Twenty runs, using a total of 20 lens cubes, were performed at each setting. For each trial, the lens cube was engaged at the phacoemulsification tip using vacuum alone. The time from the start of phacoemulsification to complete lens removal was measured using a handheld stopwatch. Only the time in which the lens fragment was engaged at the phaco tip was recorded. The time of chatter events, defined as the times in which the lens fragment dislodged from the tip, was not counted as the total time. The recorded elapsed time was considered to be the efficiency time.

The chatter events were also recorded in each trial. In this experiment, only 100% torsional power settings were used. No longitudinal ultrasound was added except for that which would be normally added as part of the Ozil-IP system. We compared efficiency and chatter events with a balanced tip and intrepid sleeve versus a kelman tip with microsmooth sleeve. One randomly chosen lens cube was placed inside a chamber filled with a balanced salt solution. The pedal was initially depressed to vacuum only for the time required to aspirate the cube to the tip. Once the lens fragment occluded the phaco tip, the pedal was fully depressed to initiate phacoemulsification. A stopwatch was used to record the time from which the particle was at the tip to the time of fragment removal, as seen under an operating microscope. The stopwatch was stopped when the particle bounced from the tip so that the chatter time was not included in the total particle removal time. The differences were compared using the Student t test or kruskall-wallis test depending on the type of comparison. Because chatter is recorded on an ordinal scale, the Wilcoxon signed-rank test was used for these comparisons.

## Results

### Experiment 1 (Temperature profile)

At the longitudinal 40% setting, there were no significant differences among the four groups. At the longitudinal 70% setting, the bare tip groups (1 and 2) and the sleeved groups (3 and 4) showed significant difference in temperature (P = 0.021; Table 1).

**Table 1:** Temperature at the incision site at a70% longitudinal setting.

	Mean Incision Temperature (°C)			
	Baseline	Elevation	Maximum	Corneal burn
Kelman(bare tip)	22.4	13	35.4	no
Balanced(bare tip)	22.1	15.5	37.6	no
Kelman(+sleeve)	22.2	23.5	46.7	yes
Balanced(+new sleeve)	22.3	21.9	45.2	yes
		Bare tip vs Sleeved tip	Group 1 vs Group 2	Group 3 vs Group 4
P—value		0.021	0.763	0.896

**Fig 3:**
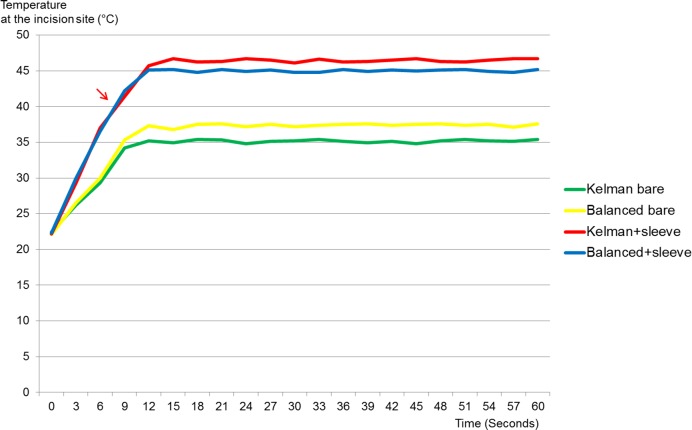
Temperature change during phacoemulsification. Temperature increase over time at 70% longitudinal power. Arrows indicate thestart point of the corneal burn.

Groups 3 and 4 produced corneal burns. A corneal burn was observed when the temperature increased above 40°C (Fig 3).

At the torsional 40% setting, there were no significant differences among the four groups. At the torsional 100% setting, in comparing the sleeved groups, we observed a lower increase in temperature in the group with balanced tip with intrepid sleeve set (P = 0.007; Table 2). No corneal burns were detected at this setting.

**Table 2:** Temperature at the incision site at a100% torsional setting.

	Mean Incision Temperature (°C)			
	Baseline	Elevation	Maximum	Corneal burn
Kelman(bare tip)	22.4	10.4	32.8	no
Balanced(bare tip)	22.2	11	33.2	no
Kelman(+sleeve)	22.1	16.6	38.7	no
Balanced(+new sleeve)	21.8	14	35.8	no
		Bare tip vs Sleeved tip	Group 1 vs Group 2	Group 3 vs Group 4
P—value		0.327	0.756	0.007

### Experiment 2 (Efficiency and chatter)

**Fig 4:**
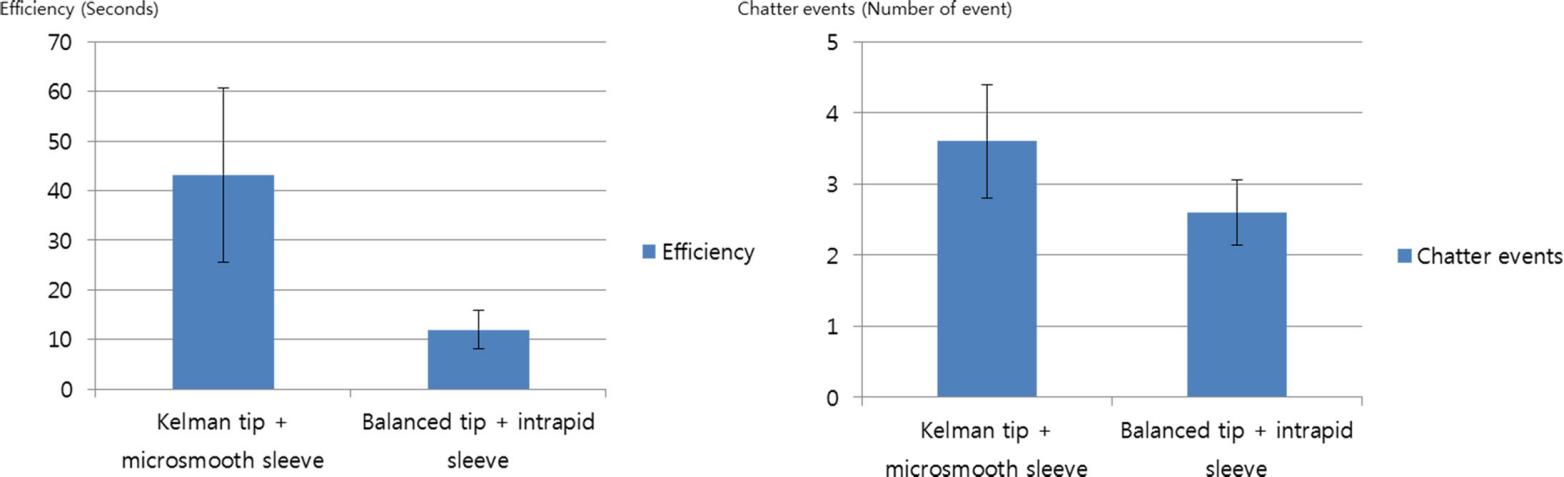
Efficiency and chatter events. A. The balanced tip shows short efficiency times at 100% torsional power. B. Chatter events show no statistical differences between the two phaco tips.

At the 100% torsional setting, the efficiencies of the balanced tip with intrepid sleeve and of the Kelman tip with mirosmooth sleeve were 13.52± 2.60 seconds and 44.45 ± 14.75 seconds, respectively (P<0.01). With the same settings, the chatter events in the balanced tip with intrepid sleeves and in the Kelman tip with microsmooth group were 2.33± 0.88 and 2.61± 1.00, respectively (P = 0.436; Fig 4).

## Discussion

Recent developments in cataract surgery technology have focused on reducing the incision size to a minimum. This provides a safer surgery with almost instantaneous recovery, low postoperative astigmatism, high predictability of postsurgical refraction. With smaller incision sizes, the irrigation tubing may not be able to provide enough fluid to cool the corneal tissue. In our present study, we could confirm significant heat production during phacoemulsification. This phenomenon is also significant in longitudinal settings, as shown in a previous publication.[8] Another earlier study, evaluating the thermal characterization of phacoemulsification probes,[9] suggested that internal metal stress and tip-to sleeve friction are two major factors in heat generation during phacoemulsification. Consistently, in our current study, tip-to sleeve friction had the greatest effect on heat generation. Indeed, the new balanced tip showed no benefit in terms of wound temperature if bare tips were compared. However, when the tips were used with sleeves, especially at the torsional 100% setting, the balanced tip achieved a lower increase in wound temperature. This may due to the rib-like design of the modified sleeve, allowing a flow through the rib space, thus preventing friction when the tip is caught in the incision site. In experiment 1, balanced tip with intrepid sleeve, and kelman tip with microsmooth sleeve induced corneal burn at 70% phaco power. Based on this result, if prolonged emulsification is needed, the operator should consider lower phaco power setting. Also, in experiment 1, the balanced tip with a intrepid sleeve showed less increase in temperature at the100% power torsional setting. Therefore, we planned experiment 2 to evaluate the efficacy and chatter event of the balanced tip with modified sleeve.

In experiment 2, the balanced tip with intrepid sleeve showed excellent efficiency compared with the Kelman tip with microsmooth sleeve. The porcine lenses we used in our experiments were soaked in formalin for 24hours, unlike those used in previous studies. Even if the hardness of the 24-hour soaked lens was high, the efficiency of the new modified phaco tip was excellent compared with the previous Kelman tip. These results may highlight the importance of the newly released balanced tip. The balanced tip was engineered with an enhanced radius of rotation, and we could confirm this efficiency as we detected a lower increase in temperature and less movement in the same density cataract.

Limitations of our study include the fact that only hard lenses were used for the efficiency test. However, the fact that the new balanced tip showed a good efficiency in a hard nucleus is very meaningful, and these experiments should be confirmed in normal-density cataracts.

In conclusion, there is a lower temperature increase with a balanced tip with modified sleeve when a 100% power torsional setting is used and excellent efficiency. This confirms the expectations of shorter times and lower temperatures for the balanced tip and modified sleeve in a hard nucleus.

## Supporting Information

S1 Fig New modified phaco tip and sleeve. *Left*: Balanced tip has same inner caliber as Kelman tip, however, its thickness decrease using titanum material. *Right*: Sleeve has been modified with 4 rib- like space as well
(TIF)

S2 Fig Porcine lens cutting methods. *Left*: Own cutting knife was made with acryl plate and laser cutter with 2mm interval. *Middle*: The lens was trapped between the blades, cutting knife were inserted plate slots. Right: The porcine lens is cut into 2.0 mm^3^ cubes.
(TIF)

S3 Fig Temperature change during phacoemulsification. Temperature increaseover time at 70% longitudinal power. Arrows indicate thestart point of the corneal burn.
(TIF)

S4 Fig A. **Efficiency and chatter events**. The balanced tip shows short efficiency times at 100% torsional power. B. Chatter events show no statistical differences between the two phaco tips.
(TIF)

S1 Table Temperature at the incision site at a70% longitudinal setting. (TIF)

S2 Table Temperature at the incision site at a100% torsional setting. (TIF)
