# Supplementary figures and images for: Heat Generation and Efficiency of a New Modified Phaco Tip and Sleeve
Source: PLoS One. 2016 Aug 3;11(8):e0159049. doi: 10.1371/journal.pone.0159049 (PMC4972308; doi:10.1371/journal.pone.0159049)

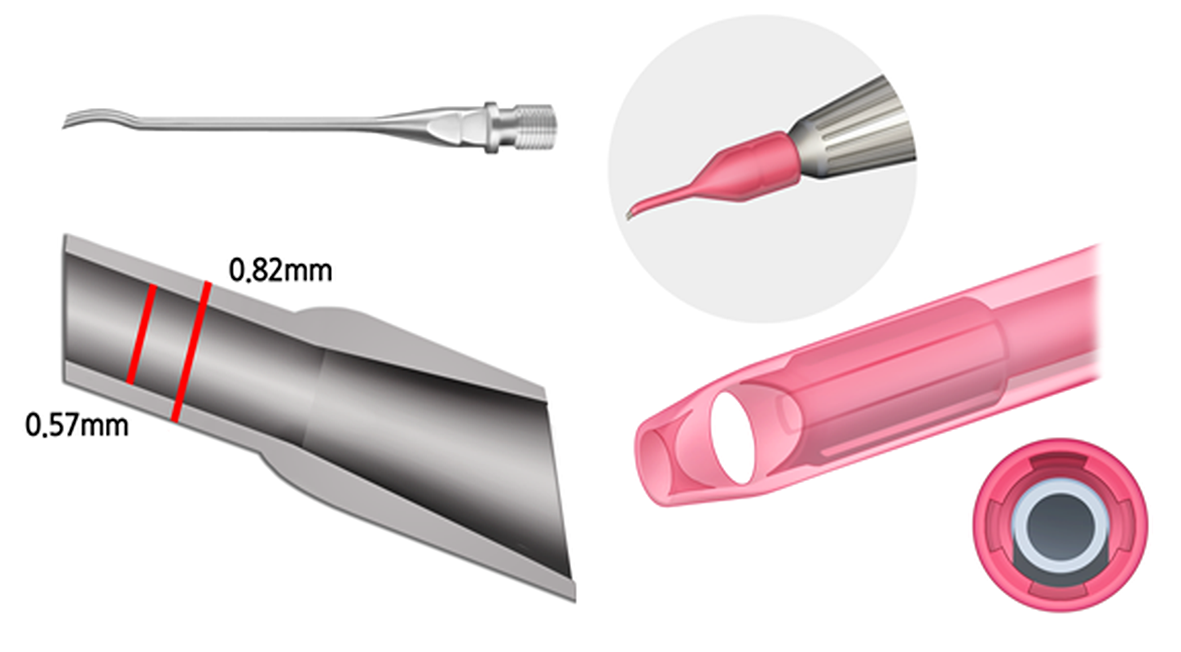

Supplement: S1 Fig — Left: Balanced tip has same inner caliber as Kelman tip, however, its thickness decrease using titanum material. Right: Sleeve has been modified with 4 rib- like space as well (TIF) [file pone.0159049.s001.tif]

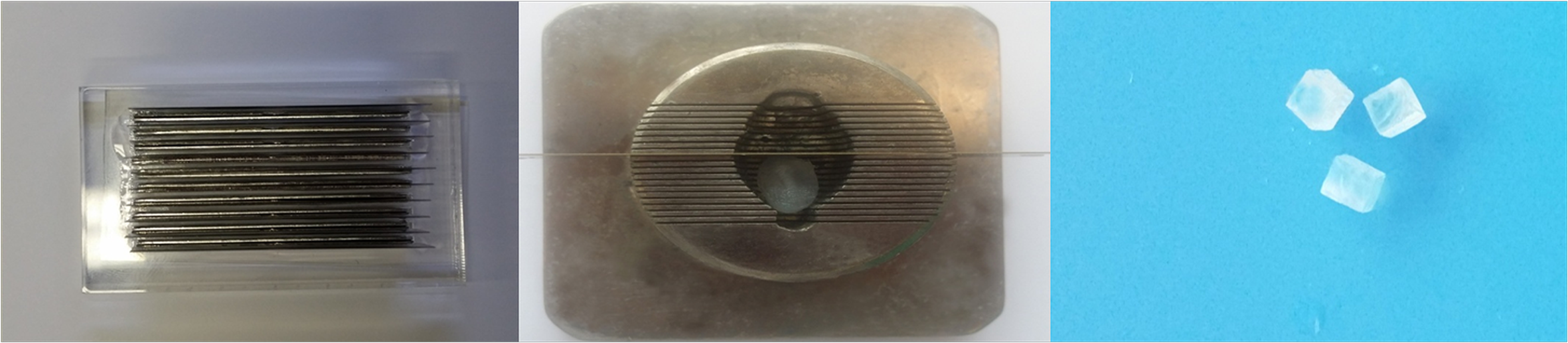

Supplement: S2 Fig — Left: Own cutting knife was made with acryl plate and laser cutter with 2mm interval. Middle: The lens was trapped between the blades, cutting knife were inserted plate slots. Right: The porcine lens is cut into 2.0 mm3 cubes. (TIF) [file pone.0159049.s002.tif]

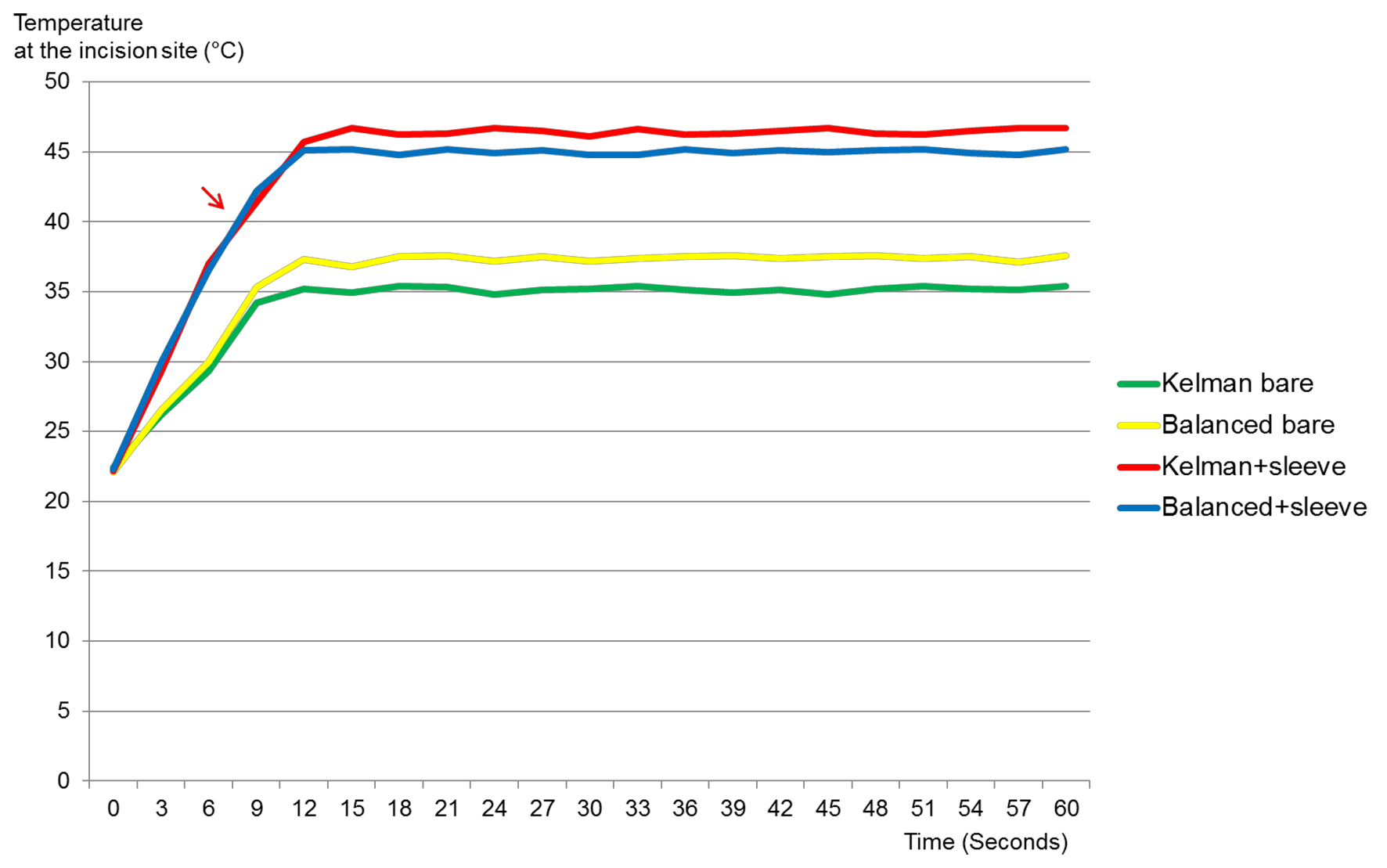

Supplement: S3 Fig — Temperature increaseover time at 70% longitudinal power. Arrows indicate thestart point of the corneal burn. (TIF) [file pone.0159049.s003.TIF]

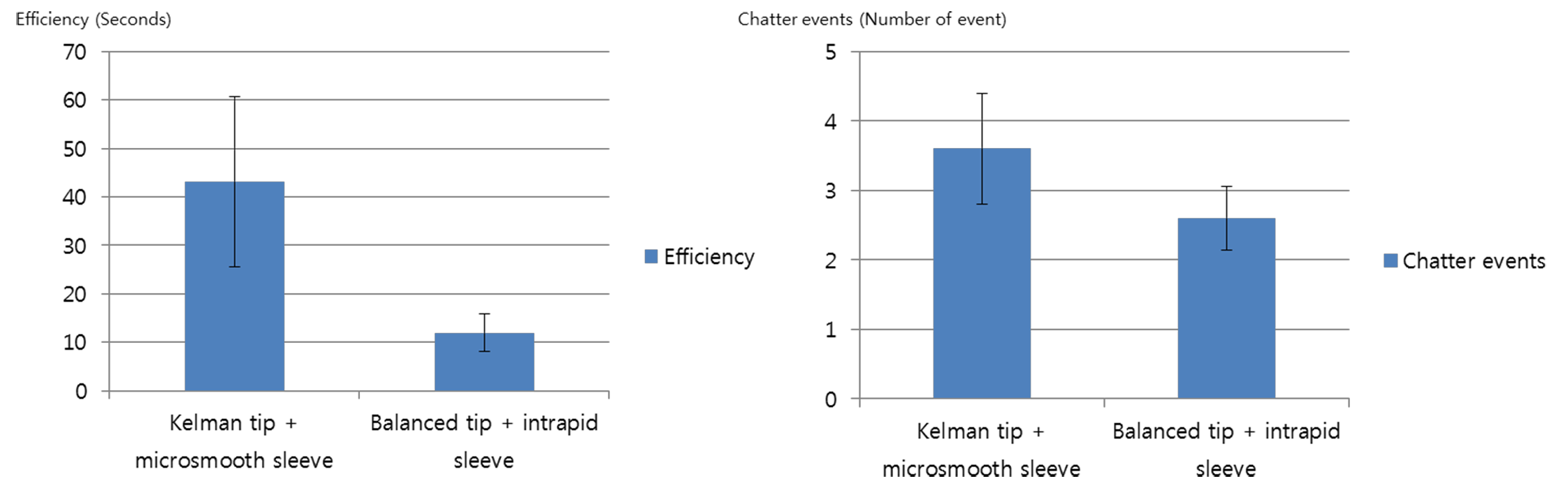

Supplement: S4 Fig — A. Efficiency and chatter events. The balanced tip shows short efficiency times at 100% torsional power. B. Chatter events show no statistical differences between the two phaco tips. (TIF) [file pone.0159049.s004.TIF]

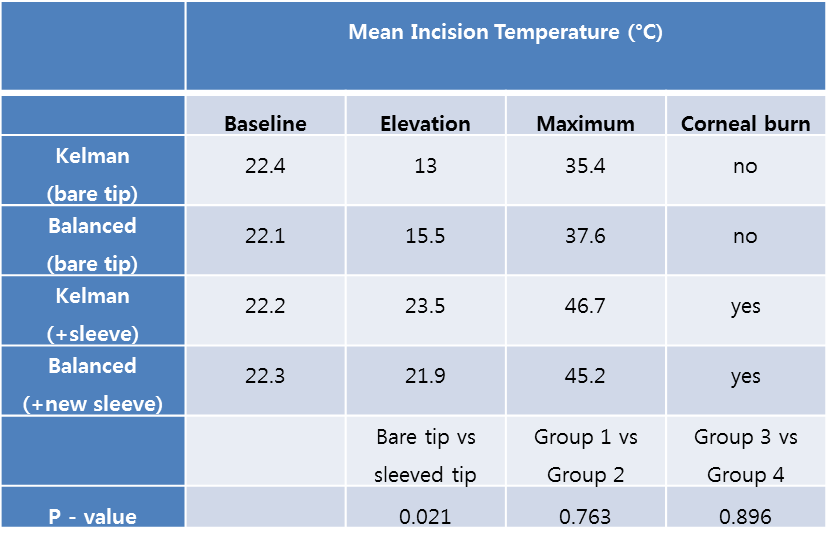

Supplement: S1 Table — (TIF) [file pone.0159049.s005.tif]

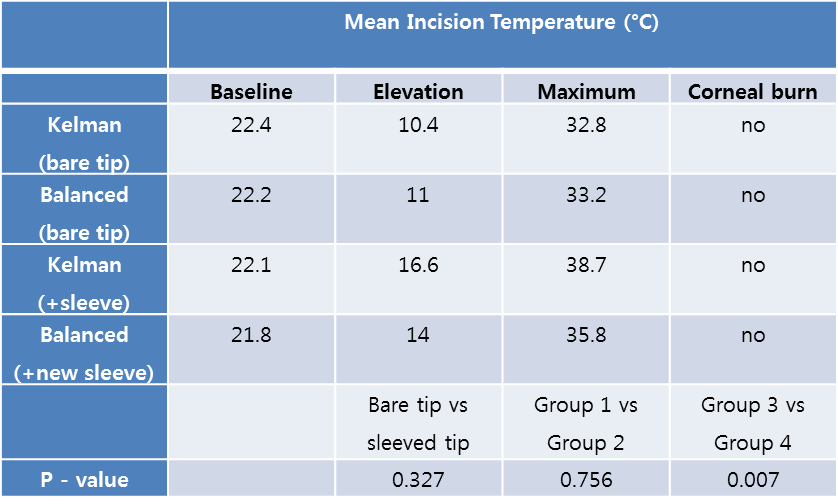

Supplement: S2 Table — (TIF) [file pone.0159049.s006.tif]
